# Can an online battery match in-person cognitive testing in providing information about age-related cortical morphology?

**DOI:** 10.1007/s11682-024-00918-2

**Published:** 2024-09-07

**Authors:** R. Thienel, L. Borne, C. Faucher, A. Behler, G. A. Robinson, J. Fripp, J. Giorgio, A. Ceslis, K. McAloney, J. Adsett, D. Galligan, N. G. Martin, M. Breakspear, M. K. Lupton

**Affiliations:** 1 School of Medicine and Public Health, The University of Newcastle, Hunter Medical Research Institute, Newcastle, NSW 2305 Australia; 2 School of Psychological Sciences, The University of Newcastle, Hunter Medical Research Institute, Newcastle, NSW 2305 Australia; 3 Queensland Brain Institute, The University of Queensland, St. Lucia, Brisbane, QLD 4072 Australia; 4 School of Psychology, The University of Queensland, St. Lucia, Brisbane, QLD 4072 Australia; 5 Australian eHealth Research Centre, CSIRO, Brisbane, QLD 4029 Australia; 6 Helen Wills Neuroscience Institute, University of California, Berkeley, CA 94720 USA; 7 QIMR Berghofer Medical Research Institute, Brisbane, QLD 4006 Australia; 8 School of Biomedical Sciences, The University of Queensland, Brisbane, QLD 4072 Australia; 9 School of Biomedical Sciences, Queensland University of Technology, Brisbane, QLD 4072 Australia

**Keywords:** Online cognitive testing, Aging, Sulcal width, Brain structure, Early detection

## Abstract

Clinical identification of early neurodegenerative changes requires an accurate and accessible characterization of brain and cognition in healthy aging. We assessed whether a brief online cognitive assessment can provide insights into brain morphology comparable to a comprehensive neuropsychological battery. In 141 healthy mid-life and older adults, we compared Creyos, a relatively brief online cognitive battery, to a comprehensive in person cognitive assessment. We used a multivariate technique to study the ability of each test to inform brain morphology as indexed by cortical sulcal width extracted from structural magnetic resonance imaging (sMRI).

We found that the online test demonstrated comparable strength of association with cortical sulcal width compared to the comprehensive in-person assessment.

These findings suggest that in our at-risk sample online assessments are comparable to the in-person assay in their association with brain morphology. With their cost effectiveness, online cognitive testing could lead to more equitable early detection and intervention for neurodegenerative diseases.

**Supplementary Information:**

The online version contains supplementary material available at 10.1007/s11682-024-00918-2.

## Introduction

Aging is associated with substantial changes in cognition (Murman, 2015) and brain structure (Lockhart & DeCarli, 2014). The trajectories of these age-related changes show significant individual differences with major clinical implications (Oschwald et al., 2020) such as early detection of neurodegenerative disorders (e.g., Alzheimer’s Disease). Comprehensive in-person multi-domain neuropsychological assessments are considered the gold standard for estimating cognitive status in research and clinical settings. This type of assessment is labor intensive, costly and not always feasible. Accessibility issues are a significant barrier for older adults to receive health care (Gaans & Dent, 2018). Recent events, such as the Covid-19 pandemic, have also been found to influence older adults’ access to health care (Bastani et al., 2021) raising the importance of reducing barriers to participation in clinical care and research.

Self-administered online cognitive testing offers several advantages over in person assessments, including greater flexibility, the ability to record accuracy and speed of response with high precision, and better cost-efficiency (Bauer et al., 2012). The popularity of online neuropsychological tests is rapidly increasing, with the availability of online cognitive batteries having more than doubled in the past decade (Mackin et al., 2018; Wild et al., 2008) and large biomedical databases such as the UK biobank (https://www.ukbiobank.ac.uk/) solely relying on computerized testing. Earlier studies initially expressed scepticism about the use of computerized testing, particularly regarding the introduction of environmental confounds and the lack of supervision (Gosling et al., 2004; Kraut et al., 2004). Nonetheless, research in large samples has shown a strong correlation (Pearson’s *r* = 0.80) between in-person and web-based cognitive testing (Germine & Hooker, 2011; Haworth et al., 2007), suggesting potential for high-quality data comparable to in-person testing when quality insurance measures are met. This is excellent considering that test-retest reliabilities of widely used in person neuropsychological tests are highly variable (ranging between *r* = 0.5–0.9 for individual tests, with memory and executive functioning scores often less than *r* = 0.7) (Calamia et al., 2013). Few studies have validated the use of online cognitive testing in older adults, but unsupervised web-based tests, including the Stroop task, paired associates learning, and verbal and matrix reasoning, have been shown to yield comparable results to supervised tests administered in a laboratory (Cyr et al., 2021). Moreover, performance on web-based tests does not appear to be correlated with technology familiarity, an issue previously raised as a potential barrier (Cyr et al., 2021).

Creyos (previously Cambridge Brain Sciences, CBS), is a widely used online cognitive assessment platform that consists of 12 self-administered tasks, based on well-validated neuropsychological tests adapted for use in a home environment (Hampshire et al., 2012). Difficulty-levels of the tasks increase with the individual’s performance level, minimising floor and ceiling effects as well as allowing for a good level of engagement. Data reliability is ensured through ‘validity’ indicators, flagging when the data are outside expected bounds. Creyos has been used in several large-scale epidemiological studies (Nichols et al., 2020, 2021; Wild et al., 2018). There have only been a limited number of studies comparing the use of the Creyos platform with in-person neuropsychological testing in older individuals (aged ≥ 40years), using small sample sizes and non-clinical populations (Brenkel et al., 2017; Sternin et al., 2019).

Cognitive changes reflect structural and functional changes in the brain. Healthy age-related changes occur in the thickness of the grey matter (cortical thickness, CT) as well as the widening of the sulci (Sulcal width, SW) (Bertoux et al., 2019), as inferred from structural magnetic resonance imaging (sMRI). SW has recently received increased attention as a robust measurement of cortical morphometry, most notably in older adults (Bertoux et al., 2019) as it appears to be less susceptible to age-related deterioration of sMRI contrast between white and grey matter (Kochunov et al., 2005). A growing number of studies suggest that greater sulcal width in older adults is associated with poorer cognitive performance and Alzheimer’s disease progression (Bertoux et al., 2019; Borne et al., 2022; Liu et al., 2011). However, whether this association is detectable with online cognitive testing remains unclear.

In this novel study, we explore the correlation between brain morphology, specifically sulcal width, and cognitive functioning across both, online and in-person modalities, providing a comprehensive examination and comparison of the relationship between brain structure and cognitive performance across both administration modalities. We first studied the mapping between online and in-person testing in a sample (*N* = 141) of healthy adults and then studied the relationship to cortical morphology as assessed with SW.

## Method

### Participants

The 141 participants (75% female, aged 46–71 years, mean age = 60; years of education: 13.1 (6.5), NART-IQ mean = 110 (SD = 9), with 50% or more completed cognitive tasks across both, the online as well as the in-person tasks were drawn from the 159 participants that had attempted online and in-person testing, within the Prospective Imaging Study of Aging (PISA) cohort- a mid-life cohort, genetically enriched for risk of AD. AD risk was defined as high or low genetic risk of Alzheimer’s disease based on Apolipoprotein E4 *(APOE* ɛ4; see Table 1) (Lupton et al., 2021). Our sample was enriched for risk for AD with 47% of participants at increased risk, compared to the general population at a 25% risk (see Table 1). Cognitive data acquired at QIMR Berghofer and structural (T1-weighted) MRI scans at Herston Imaging Research Facility (HIRF) in Brisbane, QLD, Australia (see Table 1 for a demographic overview).

**Table 1:** Participant demographics for PISA participants who completed both the online and in person cognitive assessments

Variable		Number of participants	Percent
Sex	Female	107	76%
	Male	34	24%
Education	< 12 years	38	27%
	≥ 12 years	103	73%
	Positive	66	47%
*APOE* ε4	Negative	38	38%
	Missing	21	15%

### Neuropsychological assessment

#### Online

The Creyos battery consists of 12 self-administered tasks across memory, executive function, language, and visuo-spatial domains (listed in supplementary material table S1 and fully described at https://creyos.com/). Completion of the full battery takes on average 30 min, following the guidelines of the American Psychological Association’s guidelines for the practice of telepsychology (American Psychological Association, 2023). Standardized inbuilt Creyos-provided instructions including videos and written instructions were given. Resulting scores did not require inversion before processing.

#### In-person

The comprehensive in-person cognitive battery was administered assessing cognitive domains of executive functioning, memory, language, and visuo-spatial functioning. All neuropsychological tests listed in Lupton et al. (Lupton et al., 2021) were administered by trained clinical neuropsychologists. Table 2 lists the tests that were included in this analysis. Completion of the full battery took on average two hours to complete. Where required scores were inverted so that a high score always signifies better performance (e.g., task accuracy) and a lower score indicates poorer performance (e.g., error rate, reaction time).

**Table 2:** In person cognitive battery

Domain	Task
**Memory**	Rey Auditory Verbal Learning Test - Immediate and Delayed (Ivnik et al., 1990; Rey, 1964)
	Topographical Recognition Memory Test (Warrington, 1996)
**Executive Functions**	Stroop Test (Victoria version) (Troyer et al., 2006)
	Word fluency (FAS) (Tombaugh et al., 1999)
	Digit Span F/B (Wechsler Adult Intelligence Scale - Fourth edition - WAIS-IV) (Wechsler, 2008)
	Hayling Sentence Completion Test (Burgess & Shallice, 1997) Test of Everyday Attention: Telephone Search; Dual Task (Robertson et al., 1994)
**Language**	Graded Naming Test (Warrington, 1997)
	National Adult Reading Test (Nelson & Willison, 1991)
	Spontaneous speech - complex scene description (Robinson et al., 2015)
	Category fluency – Animals (Tombaugh et al., 1999)
**Visuo-spatial**	VOSP-cube (Warrington & James, 1991)

### MRI

As part of an extensive imaging protocol, T1-weighted 3D-MPRAGE structural Magnetic Resonance Imaging (sMRI) data were acquired (TE/TR = 2.26 ms/2.3 s, TI = 0.9 s, FA = 8˚, 1 mm isotropic resolution, matrix 256 × 240 × 192, BW = 200 Hz/Px, 2x GRAPPA acceleration) at 3T on a Biograph mMR hybrid scanner (Siemens Healthineers, Erlangen, Germany). (Lupton et al., 2021).

#### APOE ɛ4 and polygenic risk score

*APOE* genotype (***ɛ***4 allele carriers vs. non-carriers) was determined from blood-extracted DNA using TaqMan SNP genotyping assays on an ABI Prism 7900HT and analysed using SDS software (Applied Biosystems). APOE *ɛ4* carriers were coded as positive across homozygous and heterozygous carriers. A polygenic risk score (PRS) to assess the overall heritable risk of developing AD was calculated by combining common AD genetic risk variants with *APOE* ɛ4 omitted (as described in Lupton et al. (Lupton et al., 2021).

#### Data processing and modelling

Python 3.11.15, with Pandas 1.2.5 and Numpy packages 1.22.4, was used throughout data processing and analyses. Specific details, including the use of other software, are included in each section. Figures were generated using packages Matplotlib 3.8.2 and Seaborn 0.11.2.

Sulcal Width (SW).

The Morphologist pipeline of the BrainVISA toolbox 4.6.0 (Borne et al., 2020) was used to extract local measures of brain anatomy from the T1-w MRI. This pipeline identifies 127 cortical sulci, 63 in the right hemisphere and 64 in the left hemisphere. Cortical thickness (CT) around each sulcus and the sulcal width (SW) were extracted; these have both shown promise for the early detection of AD. The pipeline was applied in a docker image (https://github.com/LeonieBorne/morpho-deepsulci-docker). Following Dauphinot et al. (Dauphinot et al., 2020), right and left hemisphere measurements were averaged when the same two sulci exist in each hemisphere, resulting in 64 unique measurements (see Supplementary Fig. S1 for abbreviations and labels). Docker 4.14.0, with XQuartz 2.8.5, were also used to create the SW image.

#### Partial least square (PLS)

We used a partial least square (PLS) multivariate analysis, to reduce the variables to a smaller set of predictors. PLS extracts a set of latent factors that maximize the covariance between two data sets, here cognition and cortical morphology.

First a PLS analysis was used to study the co-variation between the two cognitive assays (online and all in person test as well as online and in-person subdomains). Then, two more PLS analyses were conducted; one between the online cognitive tests and SW, and the other between the in-person cognitive tests and SW. PLS is a multivariate method that identifies modes of common variation between two data sets and ranks these according to their explained covariance. The resulting projections help identify the most important factors, often referred to as latent variables, that link the two sets of data together, to improve understanding of the relationship between them. The Canonical Partial Least Square (PLS) approach (Wegelin, 2000), implemented in the Python library scikit-learn (Pedregosa et al., 2011), was used. This method iteratively calculates pairs of latent variables (modes): the first mode corresponds to the pair explaining the most covariance, and so on for ensuing modes. These latent variables enable loadings, which weight each individual cognitive test or SW according to their contribution to that mode.

For all analyses, missing values were replaced by the average score across all participants. Sulci width features and neuropsychological measures were excluded if missing in more than 50% of participants. Likewise, participants were excluded if they were missing more than 50% of either cognitive measures or sulci width measures. In total, 3 sulci measures were excluded (F.C.L.r.sc.ant., S.GSM., S.intraCing). No participants or neuropsychological measures were excluded. All measures were z-scored by subtracting the mean of these participants and scaling to unit variance before applying the PLS.

The corresponding code is available at https://github.com/LeonieBorne/brain-cognition-pisa.

### Statistics

#### Permutation tests

Permutation tests were used to identify the robustness of the rank ordered PLS modes (Nichols & Holmes, 2002). These tests consist of randomly shuffling subject labels in one of the data domains (in this case, the cognitive measures dataset) to disrupt the empirical association with the other domain (sMRI). Then PLS is performed on these shuffled data and the covariance is measured between each pair of latent variables. This test is repeated 1000 times. If the covariance of an empirical mode is greater than 95% of those obtained from the first of these shuffled modes, then that mode is considered robust. As in Smith et al. (Smith et al., 2015); we compared scores to the first mode of the permutation tests because this extracts the highest explained variance in a null sample and can thus be viewed as the strictest measure of the null hypothesis (Wang et al., 2020).

#### Bootstrapping

Bootstrapping was used to identify which individual measures within a mode had a significant impact on the PLS latent variables (Mooney & Duval, 1993). This approach consists of creating a surrogate dataset of the same size as the original data by randomly selecting and removing participants, with replacement. This tests how robust the loadings are to particularities of the original dataset. PLS is then performed on the bootstrapped data and the loadings between each initial measure and the corresponding latent variable are calculated. This test is repeated 1000 times. If the 2.5 and 97.5 percentiles of the loadings obtained have the same sign, the measure (a specific sulcus or cognitive measure) is considered to have a statistically significant impact on the calculation of the latent variable.

### Statistical analyses

Given the strong sex-difference in AD (Zhu et al., 2021) and previous work reporting sex-differences in SW variability (Díaz-Caneja et al., 2021), we evaluated such potential sex-effects (male, female) on the relationship between in-person cognition and SW and online cognition and SW respectively using an ANCOVA, controlling for age. The strength of association between in person cognitive testing and SW versus online cognitive testing and SW was tested with Steiger’s z test. The PISA sample was enriched for high genetic risk of AD, including participants who were *APOE* ɛ4 positive, as well as those in the highest decile of risk for AD as defined by a polygenic risk score (PRS), which was calculated by combining common AD genetic risk variants with *APOE* ɛ4 omitted (as described in Lupton et al. (Lupton et al., 2021).

## Results

### Association between online performance and in-person performance

**Fig. 1:**
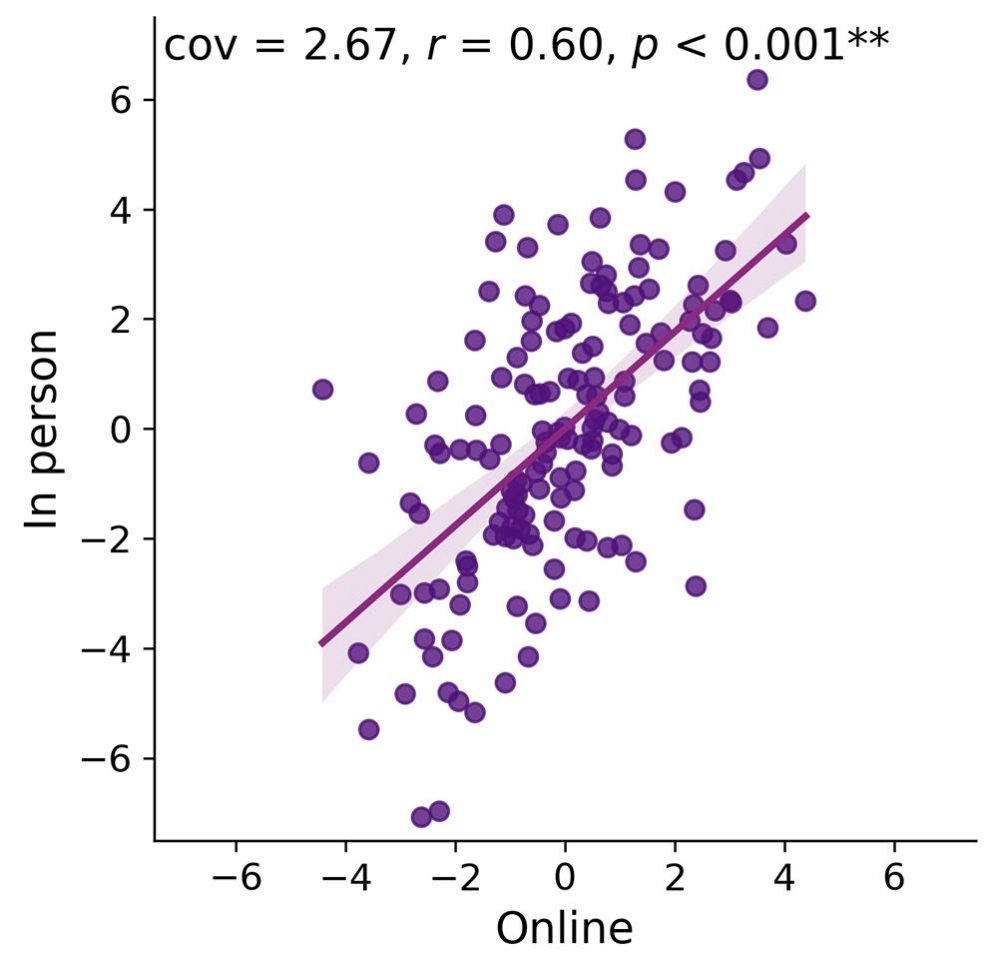
Projections of z-scored latent variables from the PLS depicting the common variation of all online tests onto all in-person tests

Across all tests, performance in online cognitive testing strongly and significantly covaried with performance in detailed in-person assessment (cov = 2.67; z-cov = 12.33; *r* = 0.60; r^2^ = 0.37; *p* < 0.001; Fig. 1).

**Fig. 2:**
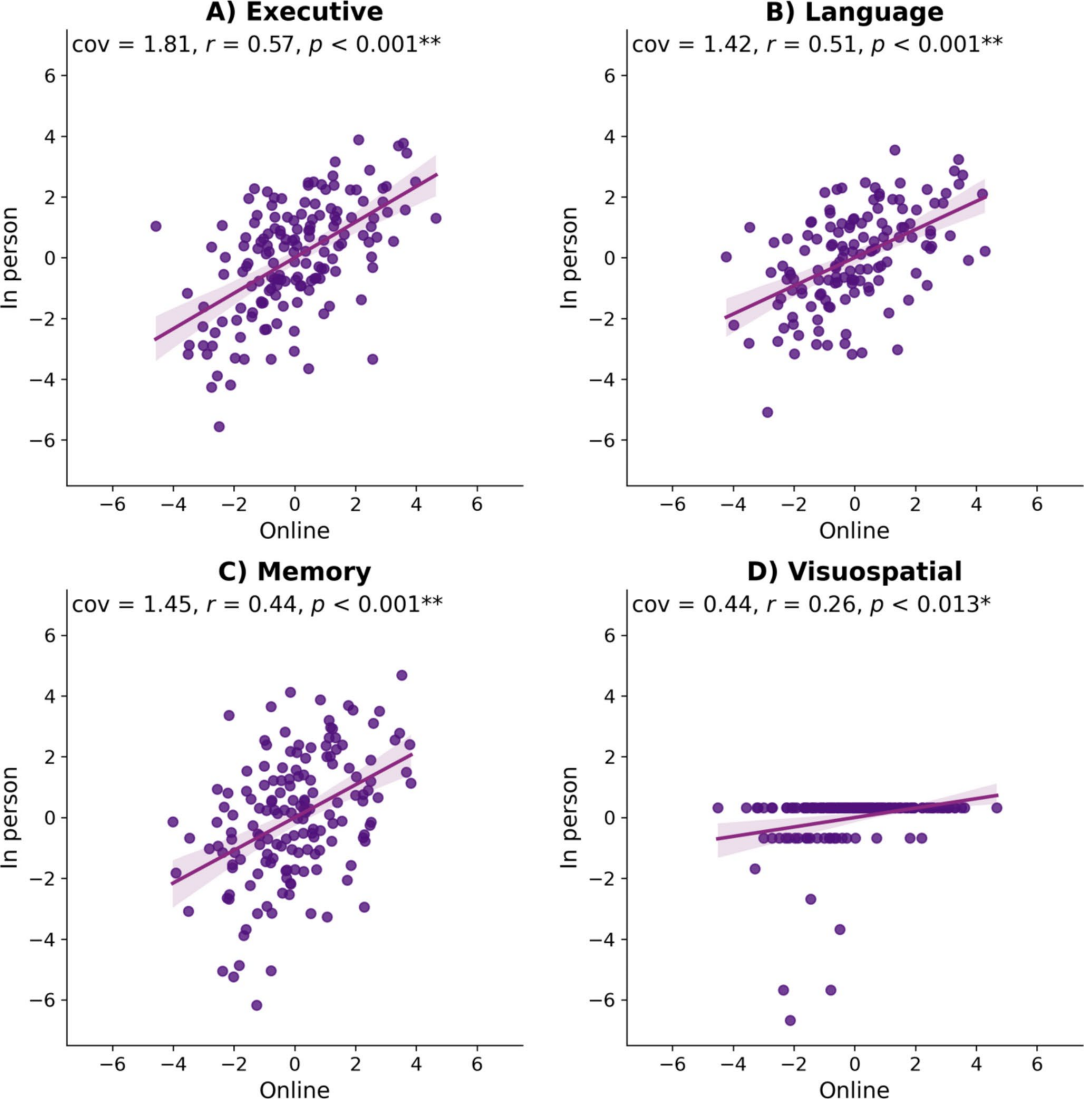
Projections (z-scored latent variables) explaining the relationship between online and onsite tests separate for the four domains (**A**-executive function; **B**-language; **C**-memory; **D**-visuo-spatial), the shaded area represents the 95% confidence interval

Analyzing different cognitive domains of the in-person assessment separately (i.e., executive, memory, language and visuo-spatial; see Fig. 2), revealed that the variance explained for executive tests of the in-person battery was strongest (cov = 1.81; z-cov = 11.57; *r* = 0.57; r^2^ = 0.32; *p* < 0.001), followed by language (cov = 1.42; z-cov = 7.09; *r* = 0.51; r^2^ = 0.26; *p* < 0.001), memory (cov = 1.45; z-cov = 6.44; *r* = 0.44; r^2^ = 0.19; *p* < 0.001), then visuo-spatial (cov = 0.44; z-cov = 2.60; *r* = 0.26; r^2^ = 0.07; *p* = 0.013). The latter task showed a ceiling effect with most participants making either no or one mistake. The average performance on the in-person and online tasks can be found in the supplementary Tables 2 and 3.

### Associations between cognition assessments and cortical morphology

**Fig. 3:**
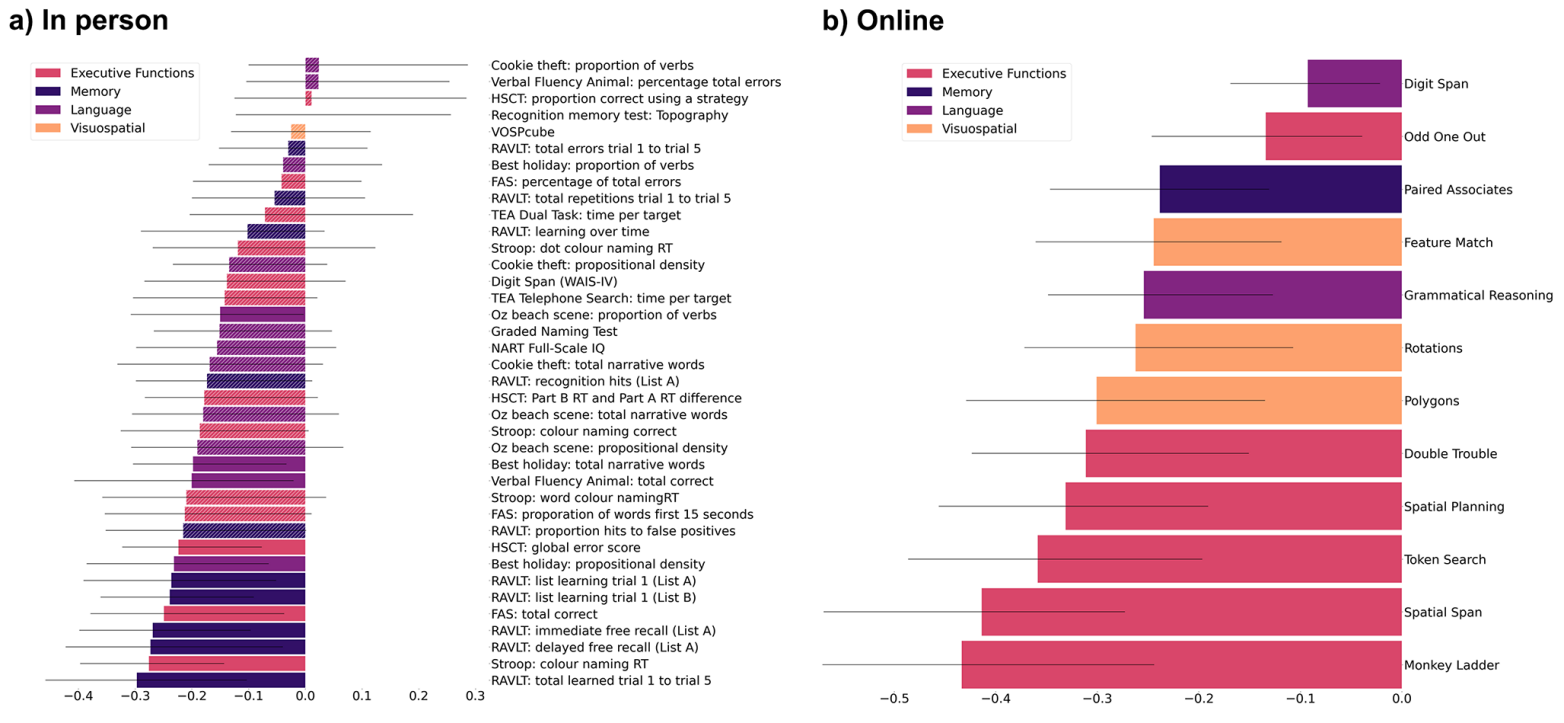
Loadings of the individual cognitive tests of the in person (left) and online (right) battery onto the latent variable of the PLS. (**a**) Cognitive test loadings for partial least square (PLS) applied to the in-person assessment, and (**b**) to the online assessment. The variables are shown in order of how strongly they load onto the latent variable, with the strongest at the bottom. Tests with non-robust associations (95% confidence intervals) are represented in bars with striped pattern

The application of partial least square (PLS) yielded a single robust mode for covariation between sulcal width and both the total online and total in-person assessments, although the nature of the loadings somewhat differed (Fig. 3). For the in-person assessment, the cognitive projection loaded most strongly onto memory and executive functions (1st mode, *p* = 0.011, cov = 3.55, z-cov = 3.00, R2 = 0.18, z-R^2^ = 0.95; 2nd mode, *p* > 0.99). For the online battery, the cognitive projection loaded most strongly onto executive function (1st mode, *p* < 0.001, cov = 2.76, z-cov = 4.71, R^2^ = 0.14, z-R^2^ = 1.15; 2nd mode, *p* = 0.99). Greater SW in these projections covaried with poorer performance in the corresponding cognitive assessments.

**Fig. 4:**
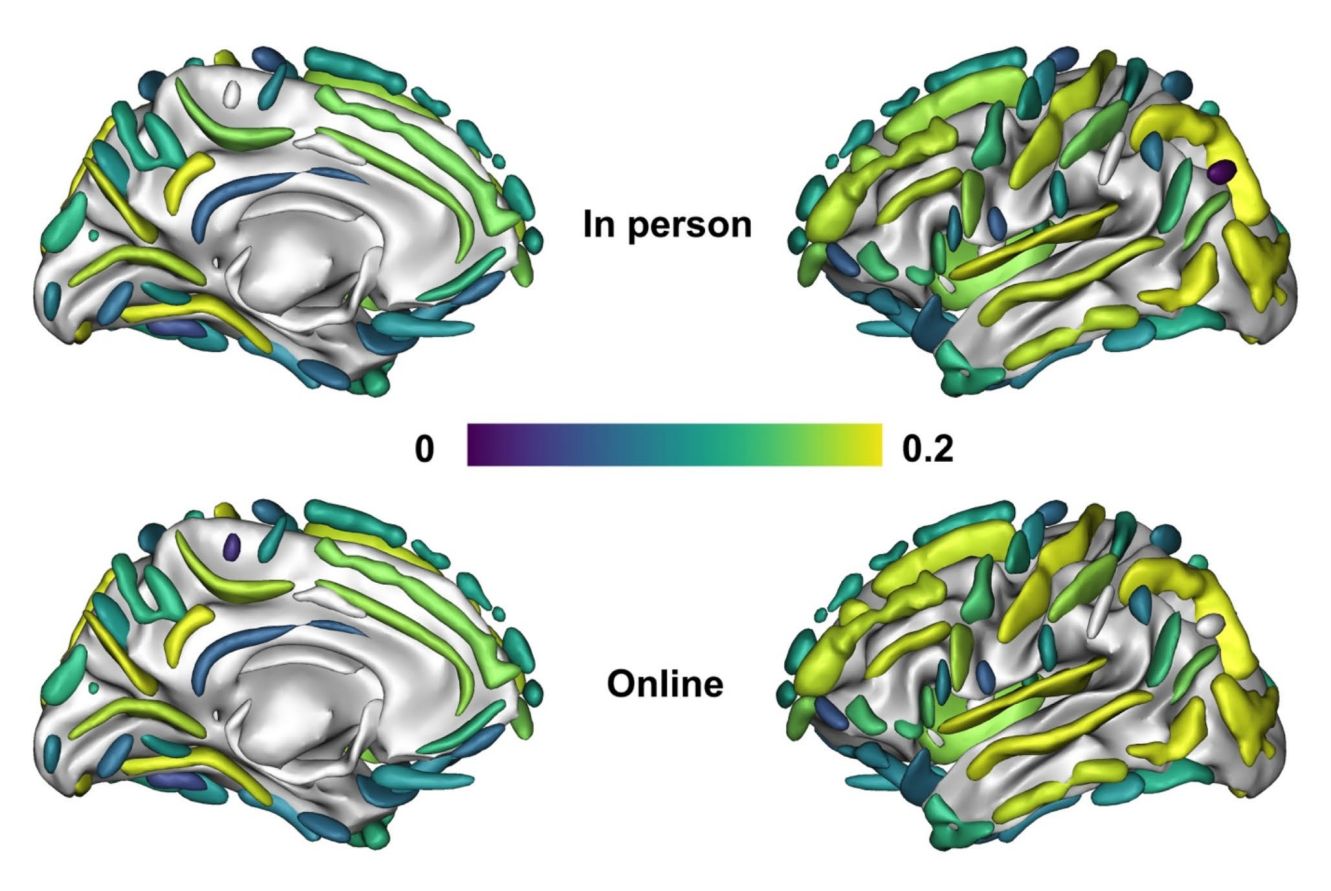
Mean loading of the in person (top) and online (bottom) latent variable onto the 127 sulci averaged across left and right hemisphere according to BrainVISA toolbox (Borne et al., 2020) for in-person (top) and online administration (bottom), with the strongest positive covariation of the latent variables of the respective cognitive assays onto the sulcal width latent variable in dark purple and the weakest association in light yellow

Brain loadings of overall cognition-related sulcal width showed a regional pattern that was significantly correlated between the online and in person cognitive appraisals (*r* = 0.996; see figure S1 in supplementary material), with both cognitive administration modalities (in-person and online) loading most strongly across the occipital lobe, the anterior and posterior inferior temporal sulcus, the posterior lateral fissure, superior, inferior and internal frontal sulcus, intraparietal sulcus, sub-parietal sulcus, and parieto-occipital fissure. Brain-behavior z-transformed covariance was likewise comparable across the two administration types (Fig. 3) with no significant difference in the variance explained in sulci width for the online cognitive assay (*r* = 0.39) compared to the in-person testing (*r* = 0.42; Steiger-z = 0.48, *p* = 0.63). Taken together both cognitive projections loaded onto similar cognitive domains and projected with comparable strength and topography onto the brain’s morphology (Fig. 4). AD risk and sex had no significant effect on the association (see Figures S2 - S4 in supplementary material).

There was no significant effect of sex on either the in-person or the online cognitive-sulcal width relationships (Fig. S4 in supplementary material).

**Fig. 5:**
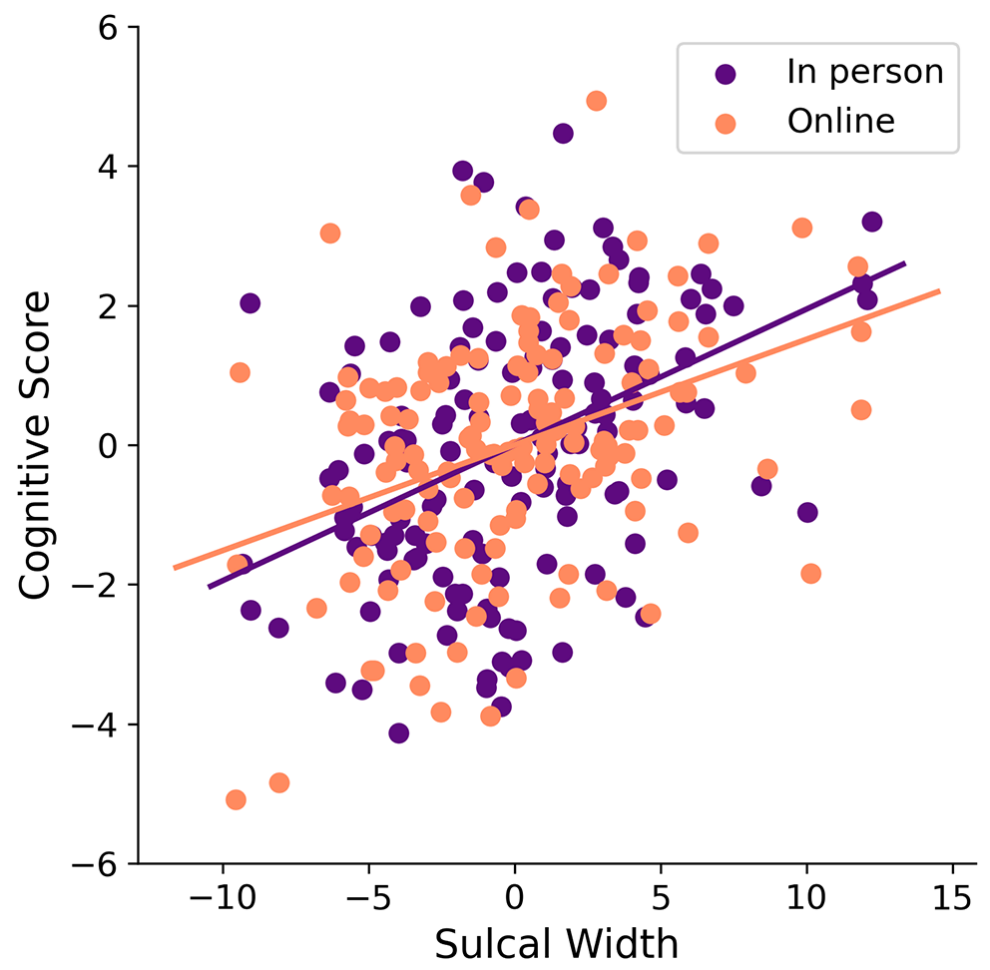
Relationship between cognition and sulcal width for in-person testing (purple) and online testing (orange) with no significant difference in the variance explained in sulci width for the two different administrations (Steiger-z = 0.48, *p* = 0.63)

There was a significant relationship between sulcal widening and cognitive performance across both online and in-person administration (Fig. 5). This association was evident regardless of age (Fig. S5).

## Discussion

With an aging population, and recent advances in treatment options in the early stages of neurodegeneration, the demand for early identification is rising. More accessible, digital cognitive testing can assist to fulfill this demand. However, such tests need to have comparable performance to traditional in person tests, and similar sensitivity to the presence and nature of underlying neurobiological differences.

Here we demonstrated that relatively brief online cognitive tests strongly co-vary with extensive in-person assessment and relate to similar underlying cortical morphology, with executive and memory domains showing the strongest loadings. This aligns with the findings of Germine and Hooker (2011) and Haworth et al. (2007), which demonstrate strong correlations between in-person and online cognitive assessments. Additionally, it reinforces other research validating the use of computer-based tests in older adults (Cyr et al., 2021). We add to this prior body of work by demonstrating that online assessments in this population produce brain projections comparable to those of in-person testing. We observed a very strong correlation between the sulcal width projections of online and in-person cognitive assays, with similarly strong variance explained for the online testing (*r* = 0.39) and the in-person testing (*r* = 0.42). By comparing online testing to in-person cognitive testing for its efficacy in informing brain morphology, we highlight its potential utility as a screening instrument in the fields of neurocognition and aging. The independence of the brain-cognition relationship from age underscores that age itself is not the sole determinant of this association. The relatively strong weighting for executive function across both in-person and online assessments is in line with West’s (1996) frontal aging hypothesis and highlights the importance of considering executive function alongside memory when investigating brain neurodegeneration in mid-life aging. In sum, the current analyses suggest adequate sensitivity of online cognitive tests for studying the age-related neurobiology of cognition.

Online cognitive testing offers cost savings, automated interpretation, accessibility, and customizable difficulty levels (Sternin et al., 2019). Our study shows a user-friendly 30-minute online platform at home correlates comparably with cortical morphology to a two-hour in-person test by a neuropsychologist.

Increasingly, online testing is employed in large-scale epidemiology studies, exemplified by our PISA study with data from over 2,000 participants (Lupton et al., 2021). In the Alzheimer’s Disease Neuroimaging Initiative (ADNI), the latest data collection wave aims to screen 20,000 participants online before further phenotyping (Weiner et al., 2022). Online testing also holds promise for assessing interventions on cognitive outcomes and serves as a screening tool for clinical trial participant inclusion (Fawns-Ritchie & Deary, 2020; LaPlume et al., 2021).

Certainly, in-person cognitive assessments present a distinct set of advantages, especially when it comes to the clinical evaluation and differentiation of various neurodegenerative disorders during their initial stages. While technology-driven cognitive assessments have their merits, the traditional in-person approach offers unique strengths that can significantly impact diagnosis and treatment, particularly in a clinical setting. Hence, while remote and technology-driven cognitive assessments have their place in modern healthcare, in-person neurocognitive assessments continue to be indispensable, especially in clinical contexts.

Current alternatives to comprehensive in-person cognitive testing include the Alternatives to in-person cognitive testing, like Mini-Mental Status Exam (MMSE (Folstein et al., 1975) and Montreal Cognitive Assessment (MoCA (Nasreddine et al., 2005), serve as screening tools for cognitive changes. Platforms like Creyos offer online testing as potential, more detailed alternatives (Sánchez Cabaco et al., 2023). Growing normative datasets may integrate these platforms into healthcare, enabling non-experts to monitor cognitive decline and assess interventions’ effects on cognition.

There are some caveats to note in the current study. The PISA cohort is enriched for those at the extremes of genetic risk for Alzheimer’s disease. This selection bias does not affect the comparison of the online versus in-person cognitive testing platforms but may predetermine the projections towards prodromal Alzheimer’s disease related impairment, rather than impairment associated with normal aging. Future validation work should also include longitudinal data to allow cognitive decline to also be assessed.

Unsupervised cognitive testing in a home environment has limitations that should be taken into account. There is the potential for incorrect use of tasks affecting the accuracy and reliability of the test results. Appropriate measures should be put in place to minimize those risks including validity checks. There is also a risk of intentional misuse such as completion by another individual or purposely failing tasks. This would need to be considered if such tests were e employed as screening tools for example for inclusion in a clinical trial.

Another limitation is that our sample consists of 75% females. This gender imbalance is a common issue in biomedical and psychological research, where females are often more likely to volunteer. This gender bias should be acknowledged when interpreting the results, as it may affect the generalizability of the findings to the broader population.

## Conclusions

Here we demonstrate that a cost efficient online cognitive battery parallels comprehensive cognitive in-person assessment in its correlation with brain morphology. This is particularly relevant given the anticipated increase cognitive screening demand resulting from recent advances in disease-modifying treatments for neurodegenerative disorders like Alzheimer’s.

## Electronic supplementary material

Below is the link to the electronic supplementary material.

Supplementary Material 1

## Data Availability

Following completion of each wave (baseline, follow-up) and appropriate quality control, de-identified PISA data will be made available to other research groups upon request. Due to privacy, confidentiality and constraints imposed by the local Human Research Ethics Committee, a “Data Sharing Agreement” will be required before data will be released. Due to ethics constraints, data will be shared on a project-specific basis. Depending on the nature of the data requested, evidence of local ethics approval may be required.
