# Supplementary material for: Can an online battery match in-person cognitive testing in providing information about age-related cortical morphology?
Source: Brain Imaging Behav. 2024 Sep 7;18(5):1215–25. doi: 10.1007/s11682-024-00918-2 (PMC11582305; doi:10.1007/s11682-024-00918-2)
Supplement: Supplementary file 1 — Supplementary Material 1 [file 11682_2024_918_MOESM1_ESM.docx]

**Supplementary Material**

Table S1.: Creyos (Previously Cambridge Brain Sciences) online battery

*Descriptions of tests have been amended from Hampshire et al. (2012) Supplemental information.*

| Domain |  | Description |
| --- | --- | --- |
| Memory | Paired Associates | Based on a test commonly used to assess memory impairments in aging clinical populations. Sets of boxes are displayed at random locations on grid. The boxes open one after another to reveal an icon, after which they close. The icons are then displayed sequentially in the centre of the screen, and the participant must select box that contained that icon. If the participant remembers all the icon‐location pairs correctly, then the next trial will have one more box. If an error is made the next trial has one less box. The test ends after three errors. The participant’s score is the maximum number of pairs successfully remembered. |
| Visuo-spatial | Rotations | Measures the ability to spatially manipulate objects in mind. On each trial, two groups of coloured squares (each with N squares) are displayed beside each other. One of the groups is rotated by a multiple of 90 degrees. The groups are either identical (when un‐rotated) or differ by the position of just one item, and participants must indicate if the groups match. They have 90 seconds to complete as many trials as possible. A correct response increases the final score by N, and the subsequent trial has groups of N+1 squares. If the response is incorrect, the total score decreases by N, and next trial has groups of N‐1 squares. |
|  | Polygons | Based on the Interlocking Pentagons task. On each trial, two overlapping wire‐framed polygons are displayed on the left side of screen, and participants must indicate whether the shape to the right is identical to one of the two overlapping ones. A correct response increases the total score by the difficulty level, and the subsequent trial will be more difficult (i.e., differences between polygons will be subtler). An incorrect response decreases the total score by the difficulty level, and the next trial will be slightly easier. |
|  | Feature Match | Based on classic feature search tasks used to measure attentional processing. In each trial, two groups of items (each with N items) are displayed beside each other. The groups are either identical in their contents (and item positions), or differ by just one item. Participants have 90 seconds to complete as many trials as possible, indicating whether the groups match. A correct response increases the final score by N, and the subsequent trial has groups of N+1 items. If the response is incorrect, the total score decreases by N, and next trial has groups of N‐1 items. |
| Language | Grammatical Reasoning | Based on Alan Baddeley’s three minute grammatical reasoning test. On each trial, a written statement regarding two shapes is displayed on the screen, and the participant must indicate whether it correctly describes the shapes pictured below. The participant has 90 seconds to complete as many trials as possible. A correct response increases the total score by one point, and an incorrect response decreases the score by one point. |
|  | Digit Span | Based on the verbal working memory component of the WAIS‐R intelligence test but only the forward version which is based on the phonological loop of working memory. A sequence of digits is displayed, one at a time, in the centre of the screen. Participants must then repeat the sequence of digits by selecting them on the on‐screen keyboard. Difficulty is dynamically varied, and the test ends after three mistakes. The resulting score is the length of the longest digit sequence successfully remembered. |
| Executive function | Token Search | Based on a test used to measure strategy during search behaviour. A set of boxes, one of which contains a hidden token, is displayed on a grid. Participants must find the token by clicking the boxes one at a time. Once found, it is hidden within another box. The token will not appear within the same box twice, thus the participant must search the boxes until the token has been found once within each box. An error is made if the participant checks a box that has: 1) already been clicked while trying to find the token, or 2) previously contained the token. If the participant makes an error, a new trial begins with one less box to search. If the token is found once in each box without any errors being made, a new trial begins with one more box to search. The test finishes after three errors. The resulting score is the maximum level completed. |
|  | Double Trouble | Variant of the Stroop test. Either the word “RED” or “BLUE” is displayed on the screen in either the colour red or the colour blue. The participant must select the probe word that correctly describes the colour of the target word. Participants have 90 seconds to complete as many trials as possible. A correct response increases the total score by one, and an incorrect response decreases the score by one. |
|  | Odd One Out | Based on a sub‐set of problems from the Cattell Culture Fair Intelligence Test. Nine groups of coloured shapes are displayed in a grid. The features define each group (colour, shape, # of items) and are related to each other according to a set of rules. Participants must deduce the rules and select the group whose contents do not correspond to those rules. They have 90 seconds to solve as many problems as possible, and the puzzles get progressively more difficult. A correct response increases the final score by one, whereas an incorrect response decreases the score by one. |
|  | Monkey Ladder | Based on a task from the non‐human primate literature. Numbered boxes are displayed at random locations within a grid. After a variable interval (number of squares * 900 ms) the numbers disappear leaving only the boxes. Participants must click the boxes in ascending numerical sequence. The test finishes after three errors, and the resulting score is the length of the longest sequence successfully remembered. |
|  | Spatial Span | Based on the Corsi Block Tapping Task. 16 purple boxes are displayed in a grid. A sequence of randomly selected boxes turn green one at a time (900 ms per green square). Participants must then repeat the sequence by clicking boxes in the same order. Difficulty is varied dynamically: correct responses increase the length of the next sequence by one square, and an incorrect response decreases the sequence length. The test finishes after 3 errors. The score is the length of the longest sequence successfully remembered. |
|  | Spatial Planning | Based on the Tower of London Task. Numbered beads are positioned on a tree, and the participant must relocate the beads so that they are arranged in ascending numerical order. They have three minutes to solve as many puzzles as possible, which become  progressively harder,requiring more moves and more complex planning. Trials are aborted if the participant makes more than twice the number of moves required to solve the problem. A successfully completed puzzle increases the final score by: (2 x minimum number of moves required) minus the number of moves made. |

Table S2.: Descriptives of the Online tasks

|  | N | Mean | Std. Deviation |
| --- | --- | --- | --- |
| SpatialSpan | 158 | 4.92 | 0.87 |
| Rotations | 155 | 56.67 | 33.07 |
| Polygons | 153 | 28.12 | 18.39 |
| Paired Associates | 154 | 4.34 | 0.96 |
| Digit Span | 155 | 5.95 | 1.42 |
| Feature Match | 157 | 90.20 | 23.51 |
| Spatial Planning | 145 | 11.87 | 6.66 |
| Grammatical Reasoning | 159 | 13.07 | 4.50 |
| Token Search | 157 | 6.42 | 2.17 |
| Double Trouble | 150 | 13.43 | 13.15 |
| Odd One Out | 154 | 10.29 | 3.08 |
| Monkey Ladder | 155 | 6.97 | 1.13 |

Table S3: Descriptives of the in-person tasks

|  | N | Mean | Std. Deviation |
| --- | --- | --- | --- |
| NART Full Scale IQ | 159 | 109.60 | 8.44 |
| Graded Naming Test | 159 | 21.89 | 3.40 |
| Oz beach scene total narrative words | 139 | 122.15 | 34.91 |
| Oz beach scene propositional density | 139 | 0.20 | 0.02 |
| Oz beach scene proportion of verbs | 139 | 0.40 | 0.07 |
| Best holiday total narrative words | 139 | 144.02 | 29.93 |
| Best holiday propositional density | 139 | 0.19 | 0.02 |
| Best holiday proportion of verbs | 139 | 0.45 | 0.06 |
| Cookie theft total narrative words | 140 | 154.96 | 34.44 |
| Cookie theft propositional density | 140 | 0.20 | 0.03 |
| Cookie theft proportion of verbs | 140 | 0.49 | 0.06 |
| Animal total correct | 159 | 24.60 | 5.66 |
| Animal percentage total errors | 159 | -2.94 | 3.96 |
| Stroop dot colour naming reaction time | 156 | -12.48 | 2.60 |
| Stroop word colour naming reaction time | 157 | -15.73 | 3.49 |
| Stroop colour naming reaction time | 157 | -25.16 | 6.75 |
| Stroop colour naming correct | 157 | 23.24 | 1.49 |
| HSCT RT B-A difference | 158 | -26.16 | 24.74 |
| HSCT global error score | 158 | -5.69 | 4.09 |
| HSCT proportion correct using a strategy | 158 | 0.49 | 0.25 |
| FAS total correct | 159 | 43.11 | 11.99 |
| FAS proportion of words in first 15 seconds | 159 | -0.40 | 0.07 |
| FAS percentage of total errors | 159 | -6.38 | 4.51 |
| Digit Span WAIS-IV | 159 | 19.72 | 4.02 |
| TEA Telephone Search time per target score | 156 | -3.07 | 0.66 |
| TEA Dual Task time per target score | 153 | -4.38 | 1.68 |
| RAVLT list learning trial 1 List A | 159 | 6.25 | 1.78 |
| RAVLT total learned trial 1 to 5 | 159 | 50.22 | 9.08 |
| RAVLT total errors trial 1 to 5 | 159 | -1.12 | 1.61 |
| M.RAVLT total repetitions trial 1 to 5 | 159 | 3.40 | 3.42 |
| RAVLT learning over time | 159 | 18.99 | 6.42 |
| RAVLT list learning trial 1 List B | 159 | 5.52 | 1.86 |
| RAVLT immediate free recall List A | 159 | 10.25 | 2.80 |
| RAVLT delayed free recall List A | 159 | 10.21 | 2.96 |
| RAVLT recognition memory hits List A | 159 | 13.67 | 1.46 |
| RAVLT proportion hits to false positives | 159 | 0.91 | 0.10 |
| Recognition memory test Topography | 159 | 26.08 | 2.74 |
| VOSP cube | 159 | 9.69 | 1.00 |

## **Statistics**

## Permutation tests

Permutation tests were used to identify the robustness of the rank ordered PLS modes [42]. These tests consist of randomly shuffling subject labels in one of the data domains (in this case, the cognitive measures dataset) to disrupt the empirical association with the other domain (sMRI). Then PLS is performed on these shuffled data and the covariance is measured between each pair of latent variables. This test is repeated 1000 times. If the covariance of an empirical mode is greater than 95% of those obtained from the first of these shuffled modes, then that mode is considered robust. As in Smith et al. [43]; we compared scores to the first mode of the permutation tests because this extracts the highest explained variance in a null sample and can thus be viewed as the strictest measure of the null hypothesis [44].

## Bootstrapping

Bootstrapping was used to identify which individual measures within a mode had a significant impact on the PLS latent variables [45]. This approach consists of creating a surrogate dataset of the same size as the original data by randomly selecting and removing participants, with replacement. This tests how robust the loadings are to particularities of the original dataset. PLS is then performed on the bootstrapped data and the loadings between each initial measure and the corresponding latent variable are calculated. This test is repeated 1000 times. If the 2.5 and 97.5 percentiles of the loadings obtained have the same sign, the measure (a specific sulcus or cognitive measure) is considered to have a statistically significant impact on the calculation of the latent variable.

## Mean imputation

We followed a mean imputation approach due to its relative simplicity and ease of use over alternative imputation approaches. We believe this had negligible effects in the covariance estimation at the core of the PLS estimation. Simulation studies evaluating the effect of proportion of missing variables in a PLS framework show that PLS produces unbiased estimates as long as missingness does not exceed 8.89% (DOI: 10.5445/KSP/1000098011/). Here, we had missing observations for 56 out of a possible 1908 online variables (3% missingness) and 198 out of a possible 6042 in person variables (3.2% missingness). As such, the low proportion of mean imputed variables will unlikely bias the estimation of the PLS modes presented in this manuscript.

Figure S1: Comparison of sulci-loadings online (left) and in-person (right)(English references for French sulci nomenclature is provided in the Brainvisa Sulci Atlas below)


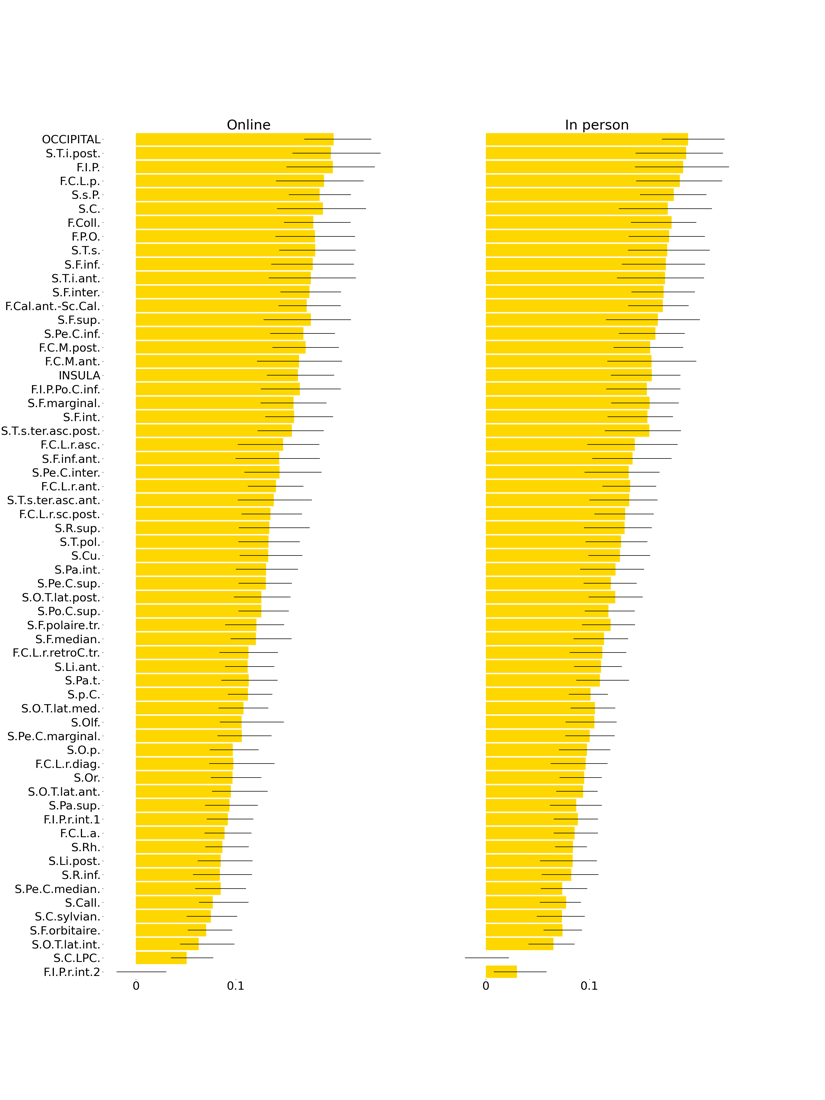


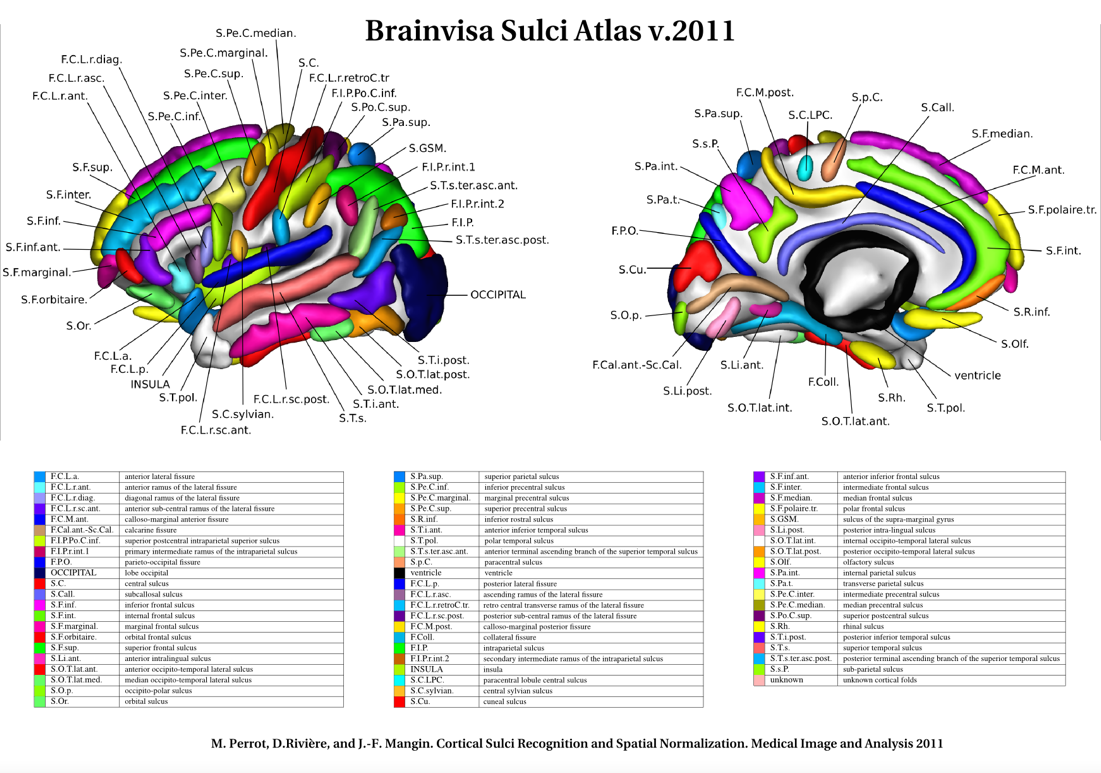


**Figure S2: Effects of *APOE* status on PLS loadings of latent variables of online cognition (top) and sulcal width (bottom). Box and whiskers show mean and inter-quartile range of
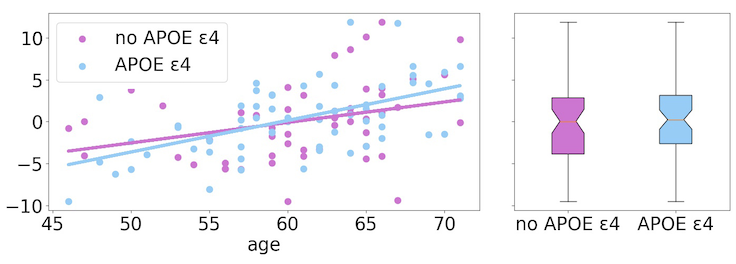

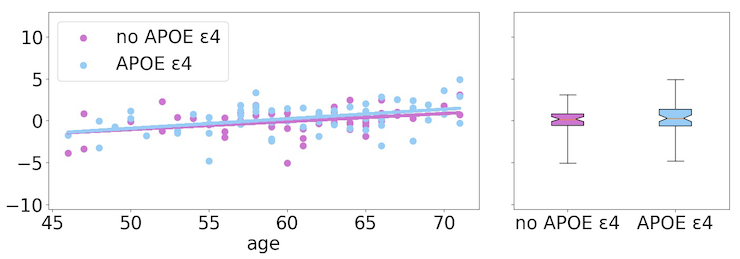
effects across ages.**

No significant effects of *APOE* ε*4* status on the cognitive projections (top) and sulcal width projections (bottom) in the online condition. Higher scores of these loadings correspond to worse task performance and wider sulci, respectively.


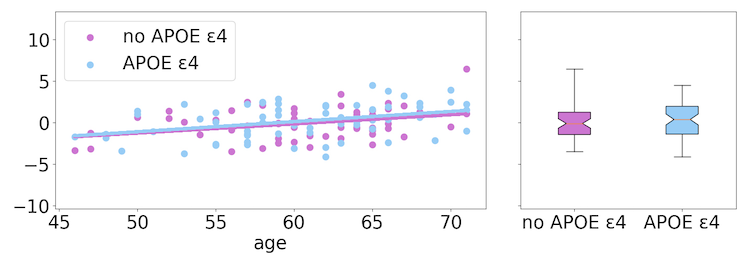
**Figure S3: Effects of *APOE ɛ4* status on PLS loadings of latent variables of in person cognition (top) and sulcal width (bottom). Box and whiskers show mean and inter-quartile range of effects across ages.**

**
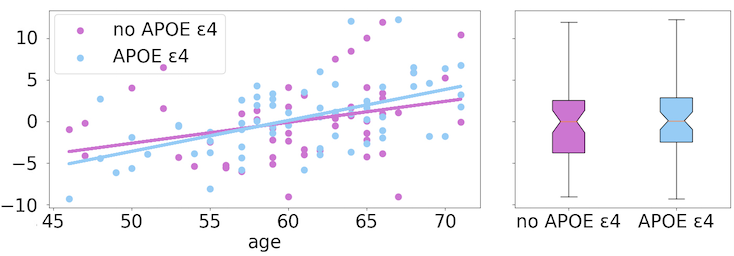
**

No significant effects of *APOE* ***ɛ****4* status on the cognitive projections (left) and sulcal width projections (right) in the in-person condition. Higher scores of these loadings correspond to worse task performance and wider sulci, respectively.

**Figure S4:** Effects of sex, on the cognition-sulcal width relationship for in-person testing (left) and online testing (right). Higher scores of these loadings correspond to poorer task performance and wider sulci, respectively.


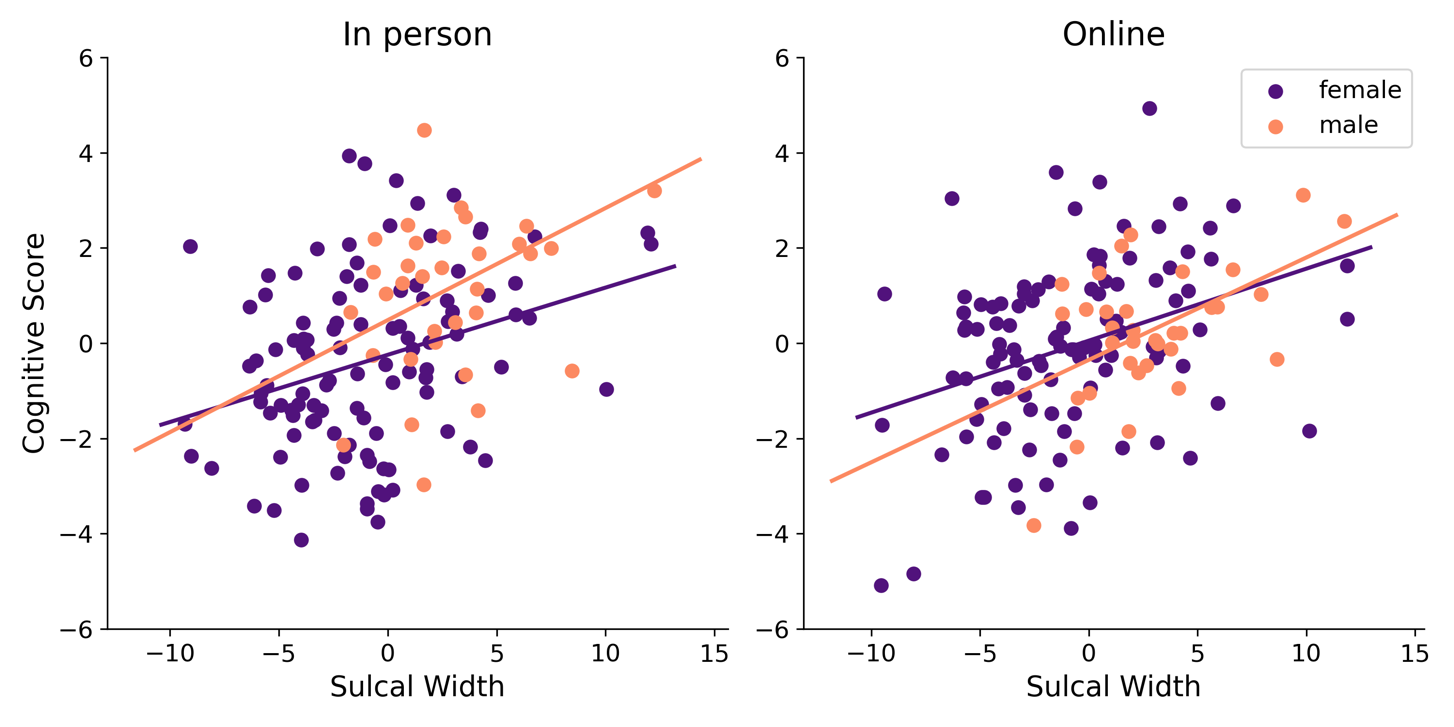


No significant sex effect on the covariance between sulcal width and either cognitive administration - in person (left) and online (right).

**Figure S5:** Effects of age (below and above 61 years), on the cognition-sulcal width relationship for in-person testing (left) and online testing (right). Higher scores of these loadings correspond to poorer task performance and wider sulci, respectively.


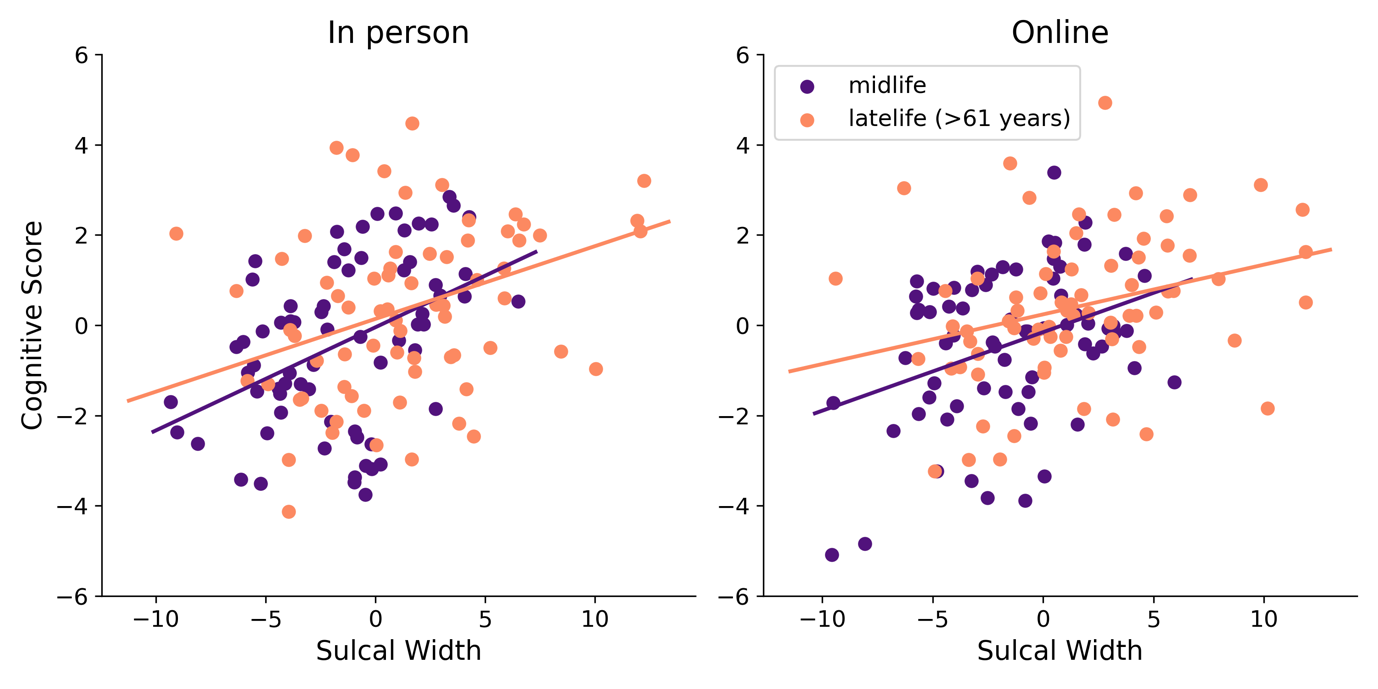


Significant relationship between cognition and sulcal width for in-person testing (left; p<0.0001) and online testing (right; p<0.0001). Effects of age (median half-split at age 61) were not significant.

Supplementary References

Hampshire, R. R. et al *Neuron* 76 (6) 1225-1237 (2012)
